# Hints on the Early Use of the Needle and Ligature in Hæmorrhages

**Published:** 1805-08-01

**Authors:** 


### Hints on the early Use of the Needle and Ligature in Hæmorrhages.

*To the Editors of the Medical and Physical Journal.*

Gentlemen,

I Accidentally observed, in No. 77 of the Medical and Physical Journal, some observations on the account which one of your Correspondents had given of the introduction or invention of the needle and ligature, by Ambrose Paré. It immediately came to my recollection, that I had long ago perused a much more complete and satisfactory account of this invention, in the first volume of Dr. Rees's New Cyclopœdia, under the articles "*Amputation*" and "*Aneurism,*" which are generally allowed to have been written by Mr. Blair. If your Correspondents, who have interested themselves in this historical investigation, will take the trouble of consulting the Cyclopœdia, they will find a number of authors referred to by Mr. Blair, who either either used the needle and ligature themselves, or described the practice of other surgeons, many years before the time of Ambrose Paré. But, while justice is done to those practitioners, in opposition to the statements of Mr. John Bell, of Edinburgh, who has treated this subject at large, the real improvements of Paré are likewise represented with candour and impartiality. In short, it is my opinion that the Editors of the Med. and Phys. Journal would confer a favour on their Readers by transcribing the "*Historical Sketch of Amputation,*" which is contained in the Cyclopœdia, as this great national work is too voluminous and expensive to fall into the hands of medical men in general; and it is perfectly clear, that both the gentlemen who have recently transmitted their remarks to the Editors of the Journal, have never seen what is contained in the Cyclopœdia upon the subject in question. Under the article "*Aneurism,*" will be found an extract from Ætius, which proves that the Greeks were acquainted with the practice (lately revived by Mr. Abernethy and others) of tying and dividing the trunk of the artery, above the aneurismal tumour.

CHIRON.

